# Closely related *Salmonella* Derby strains triggered distinct gut microbiota alteration

**DOI:** 10.1186/s13099-022-00480-6

**Published:** 2022-01-25

**Authors:** Xiaohui Yuan, Han Xue, Xiaomeng Xu, Xinan Jiao, Zhiming Pan, Yunzeng Zhang

**Affiliations:** 1 Jiangsu Co-Innovation Center for Prevention and Control of Important Animal Infectious Diseases and Zoonoses, Yangzhou University, Yangzhou, China; 2 Jiangsu Key Laboratory of Zoonosis, Yangzhou University, Yangzhou, China; 3 Key Laboratory of Prevention and Control of Biological Hazard Factors (Animal Origin) for Agrifood Safety and Quality, Ministry of Agriculture of China, Yangzhou University, Yangzhou, China; 4 Joint International Research Laboratory of Agriculture and Agri-Product Safety of the Ministry of Education, Yangzhou University, Yangzhou, China

**Keywords:** *Salmonella* Derby, Gut microbiota, Inter-strain variation

## Abstract

**Background:**

*Salmonella* Derby is one of the most predominant *Salmonella* serotypes that seriously threatens food safety. This bacterium can be further differentiated to sub-populations with different population sizes; however, whether and how the *S.* Derby–gut microbiota interactions affect epidemic patterns of *S.* Derby sub-populations remain largely unknown.

**Results:**

We selected two representative strains, 14T and 14C, which represent rarely distributed and prevalent sub-populations of the *S.* Derby ST40 group, respectively, to address this question using a mouse model. Effects of oral administration of both strains was monitored for 14 days. Alpha diversity of gut microbiota at early stages of infection (4 h post infection) was higher in 14C-treated mice and lower in 14T-treated mice compared with controls. Strain 14T triggered stronger inflammation responses but with lower pathogen titer in spleen compared with strain 14C at 14 days post infection. Certain known probiotic bacteria that can hinder colonization of *Salmonella*, such as *Bifidobacteriaceae* and *Akkermansiaceae*, exhibited increased relative abundance in 14T-treated mice compared with 14C-treated mice. Our results also demonstrated that *Ligilactobacillus* strains isolated from gut microbiota showed stronger antagonistic activity against strain 14T compared with strain 14C.

**Conclusions:**

We identified how *S*. Derby infection affected gut microbiota composition, and found that the 14T strain, which represented a rarely distributed *S*. Derby sub-population, triggered stronger host inflammation responses and gut microbiota disturbance compared with the 14C strain, which represented a prevalent *S*. Derby sub-population. This study provides novel insights on the impacts of gut microbiota on the epidemic patterns of *Salmonella* populations.

**Supplementary Information:**

The online version contains supplementary material available at 10.1186/s13099-022-00480-6.

## Background

Understanding the interactions between gut microbiota and *Salmonella* is critical to managing and preventing infection caused by *Salmonella* through microbiome engineering approaches such as probiotic administration [1, 2]. Increasing evidence has demonstrated that gut microbiota, particularly certain keystone microorganisms inside the microbiota, play an important role in the *Salmonella* colonization and infection process, and the colonization and infection process may be negatively or positively regulated by the microbiota [3–8, 22]. For instance, *Lactobacillus acidophilus* and *Mucispirillum schaedleri* are proven to ameliorate *S.* Typhimurium-induced diarrhea and inflammation [9, 10]. Conversely, certain saccharolytic bacteria, such as *Bacteroides thetaiotaomicron*, can confer advantages to *S.* Typhimurium during infection by producing 1,2-propanediol, which serves as an electron acceptor to promote respiratory growth of the pathogen [6]. These findings have shown promising potential in *Salmonella* management. However, the majority of our current knowledge on the interactions between *Salmonella* and gut microbiota comes from studies on a single well-characterized strain, such as *S.* Typhimurium ATCC 14028, or from only a few *Salmonella* serotypes including *S.* Typhimurium. Little is known about how other *Salmonella* serotypes interact with gut microbiota and the consequences of the interactions on the epidemic of the pathogens, although many serotypes have caused dramatic economical losses and threatened food safety and public health. Recent studies have demonstrated that strains affiliated with the same *Salmonella* serotype can also exhibit distinct inter-strain variations on epidemic prevalence, pathogenicity, and risk to food safety, etc. [11–15]. However, the role of gut microbiota–*Salmonella* interactions on the observed epidemic pattern differences as well as other variations among strains that harbor conserved genomic backgrounds (i.e., strains from same serotype or even same sequence type (ST) scheme) remain elusive. Here, we compared the interaction patterns between gut microbiota and two representative *S*. Derby strains that were selected from two *S*. Derby sub-populations with distinct population sizes (rarely isolated vs. frequently isolated in our multi-site, long-term epidemic investigation study) to address these questions.

Recent epidemic studies have demonstrated that *S*. Derby has become one of the predominant *Salmonella* serovars isolated from pork samples and humans globally [16–18]. For instance, around 27% of pork samples collected from China were reported to be contaminated by *S.* Derby [17]. *S.* Derby causes long-term asymptomatic infection in pigs, and can be released from the gastrointestinal tract during the slaughtering process, which is probably the main cause of the high contamination rate in the pork samples [19, 20]. More importantly, *S.* Derby has become one of the most common *Salmonella* serotypes identified in diarrheal patients in China, causing heightened interest in the pig farming industry to eradicate this pathogen [21]. Strains affiliated with the *S.* Derby serotype have been further differentiated to ST-types with the multilocus sequence typing method [14]. Of the identified *S.* Derby strains isolated from the pork production chains in China, ST40-affiliated strains were the most predominant population, and the ST40 group was further differentiated into sub-populations with distinct population sizes using a very high-resolution CRISPR-typing method [14, 15]. For example, our laboratory collected 369 ST40-affiliated strains from multiple sites in Jiangsu province during several years. These strains were differentiated into 40 sub-populations based on the CRISPR-typing method. Certain sub-populations, such as CRISPR-type 38, were found to be present in many locations and persisted throughout the sample collection course, whereas some sub-populations were rarely identified during this long-term epidemiological study (e.g., CRISPR-type 39 contained only one strain) (Additional file 1: Figure S1) [14, 15]. In the current study, we selected two representative strains, 14T (the sole strain in CRISPR-type 39) and 14C (a representative strain of CRISPR-type 38 strain group), and investigated the conserved and specifically altered patterns of gut microbiota triggered by the two strains.

## Results

### *S*. Derby 14T and 14C exhibited distinct cellular adhesion ability and triggered significantly different host inflammation response

**Fig. 1:**
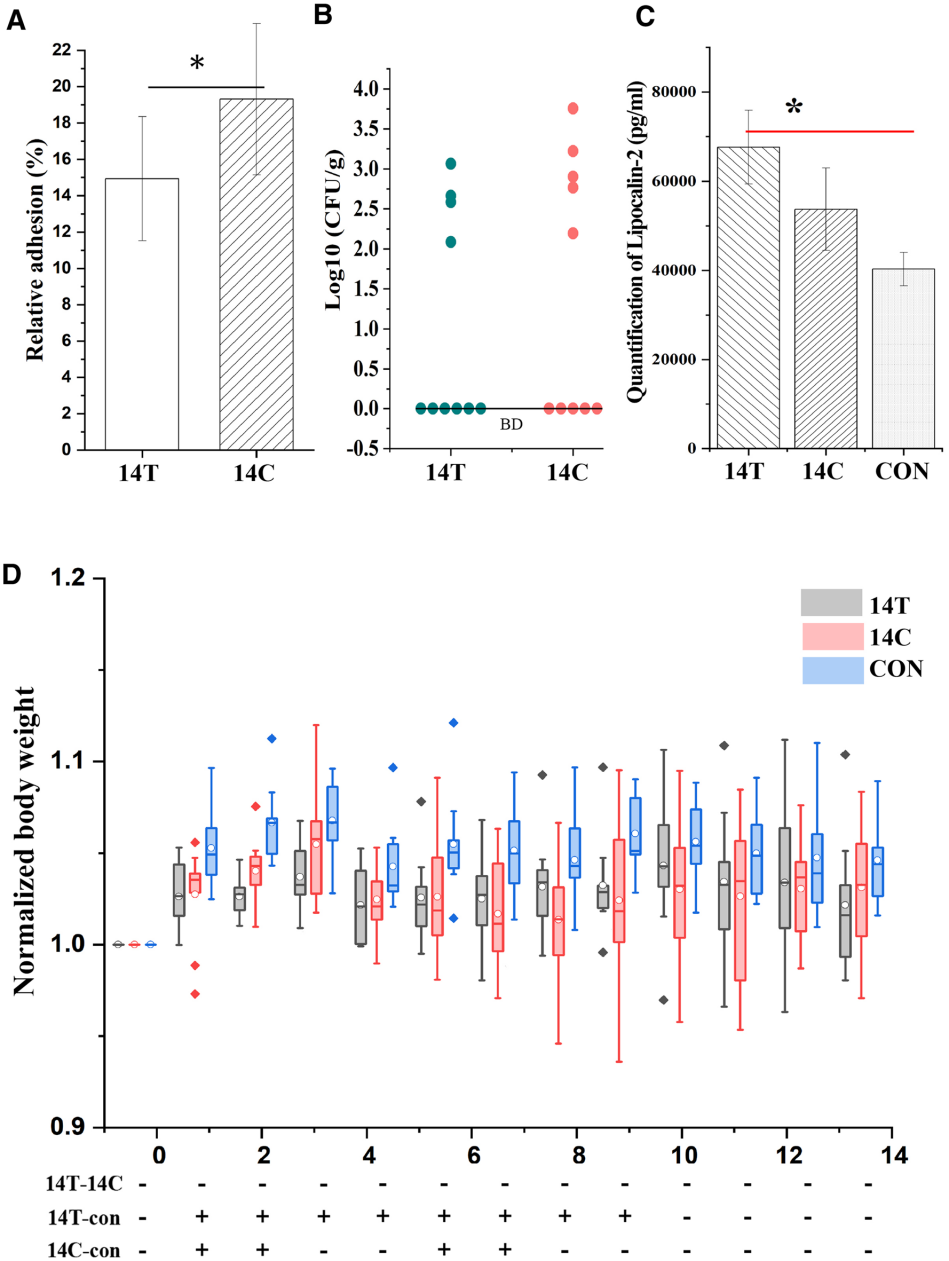
**A** Adhesion rates of *Salmonella* Derby 14T and 14C to MC 38 cells, Wilcoxon signed-rank test, *p < 0.05, error bars represent SE from mean, four independent biological replicates are included, and three technical replicates were included for each biological replicate. **B** Bacterial titers of 14T (n = 10) and 14C (n = 10) in spleen. BD, below the detection limit. **C** Quantification of lipocalin-2 level in serum at 14 days post infection among treatments (n = 9 for 14T and 14C treatments and n = 10 for control group), one-way ANOVA with post-hoc Tukey HSD test, *p < 0.05. **D** Body weight of mice at different time points among treatments (n = 10 for 14T and 14C treatments and n = 9 for control group). The body weight difference between 14T and 14C treatments were determined using one-way ANOVA with post-hoc Duncan test, with significant differences were labeled with + (*p* < 0.05)

In our previous study, we isolated 369 *S*. Derby ST40 strains in Jiangsu, China during 2011–2016, and subtyped these strains to 40 sub-populations using a CRISPR typing profiling analysis [14, 15]. We selected representative strains from each sub-population and determined host responses to pathogen infection using the mouse model. Interestingly, we observed that two closely related strains, 14T and 14C, showed distinct cellular adhesion ability and triggered significantly different host inflammation response (Fig. 1). Of note, 14T was the sole strain representing the CRISPR type 39, while 14C was a representative strain from a long-term existing sub-population CRISPR type 38 that contained multiple strains isolated from 2011–2015 (Additional file 1: Figure S1) [15]. Strain 14C exhibited significantly higher epithelia cell adhesion than strain 14T (19. 26 ± 4.14% for 14C and 14.94 ± 3.41% for 14T, mean ± SE, four independent biological replicates, Wilcoxon signed-rank test, *p* < 0.05) (Fig. 1A). When determined at 14 days post infection (dpi), the bacterial titer in spleen of the 14C-administrated mice was higher, although not significantly higher, than that of the 14T-administrated mice (Mann Whitney test, *p* > 0.05) (Fig. 1B). Interestingly, both 14T- and 14C-treated mice exhibited higher Lipocalin-2 levels compared with control, and those treated with 14T exhibited significantly stronger inflammation response compared with 14C (one-way ANOVA with post-hoc Tukey HSD test, *p* > 0.05) (Fig. 1C). Furthermore, we found that the mice treated with 14T and 14C gained less body weight compared with the control mice at several time points (one-way ANOVA with post-hoc Duncan test, p < 0.05), and the result also suggested 14T-treated mice gained less body weight than the 14C-treated mice at 2 dpi and 3 dpi, despite the difference was not significant (Fig. 1D).

### Strains 14T and 14C perturbed the gut microbiota in different manners

To reveal the roles of gut microbiota on the observed different host inflammation response as well as other differences triggered by the two *S*. Derby strains, we sought to select representative time points in the infection course based on the in vivo* S*. Derby population dynamic (Additional file 1: Table S1) for gut microbiota comparison analysis. Finally, we select three representative time points: 0 dpi (approximately 4 h post infection when high population of *S.* Derby strains have existed in the gut), 2 dpi (*S.* Derby has successfully invaded the spleen and liver), and 14 dpi (majority of the invading *S.* Derby strains have been eliminated from the gut), and collected the fecal samples for 16S rDNA V4 region sequencing analysis.

A total of 5,446,132 high quality reads were generated from 87 fecal samples, and the average number of sequences per sample was 62,599 (SD, 6308), with highest number 72,646 and lowest number 33,869 per sample. These sequenced were trimmed, filtered and clustered into 1491 Operational Taxonomic Units (OTUs). The rarefaction curve demonstrated that the OTU number increased gradually and tended to be flat as the depth of sequencing increased (Additional file 1: Figure S2), which suggested that our sequencing had nearly saturated and the abundance results could reflect the bacterial diversity in the samples confidently.

**Fig. 2:**
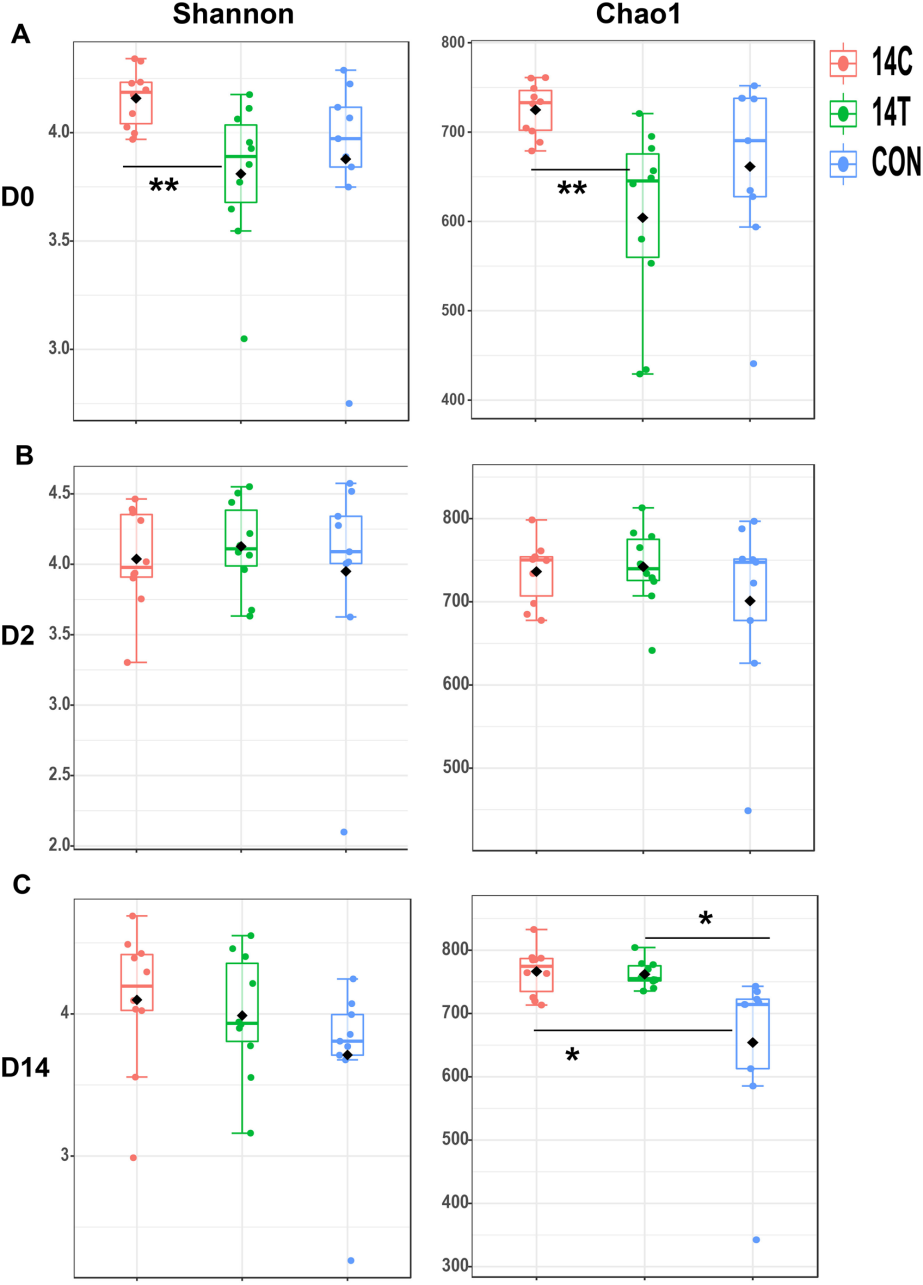
Shannon and Chaol diversity indices of 14T, 14C, and untreated control mice at **A** 0 days post infection (dpi), **B** 2 dpi, and **C** 14 dpi. Mean values are denoted as black diamonds. *p < 0.05, **p < 0.01, according to one-way ANOVA with post-hoc Tukey HSD test. At each timepoint, ten samples from 14T and 14C treatments, and nine samples from the control group were included

The alpha diversity of gut microbiota in 14T- and 14C- administrated mice was measured using Shannon and Chao1 indices. Both indices suggested that the microbial diversity and richness was significantly lower in the 14T-treated mice than in the 14C-treated mice at 0 dpi (one-way ANOVA with post-hoc Tukey HSD test, *p* < 0.005) (Fig. 2A), with the difference diminishing over time. No significant differences among the 14T, 14C and control groups were observed when tested at 2 dpi (ANOVA, *p* > 0.05) (Fig. 2B). The Chao1 index of both 14T and 14C groups was significantly higher compared with the control group at 14 dpi, suggested that the *S*. Derby treatment dramatically increased the species richness of the microbial community, while the microbial diversity was not significantly different among the three groups as suggested by the Shannon index (Fig. 2C).

The beta diversity results showed that the 14T administration triggered dramatical microbial community alteration at 0 dpi (PERMANOVA, *p* < 0.05), while the influence of 14C administration on the gut microbial community structure was not significant (PERMANOVA, *p* > 0.05) (Additional file 1: Figure S3). As a result, the community structure between the 14T and 14C groups differed significantly (PERMANOVA, *p* < 0.005) (Additional file 1: Figure S3). The community structure of the 14T-treated mice was significantly different from that of the control mice at 2 dpi (PERMANOVA, *p* < 0.05), while the community structure difference was not significant between 14C and control groups or 14T and 14C groups. No significant community structure difference was observed among the three groups at 14 dpi (PERMANOVA, *p* > 0.05) (Additional file 1: Figure S3). Taken together, these results suggested that 14C administration did not altered overall structure of the gut microbiota during the infection course, while the community structure was significantly altered by the 14T strain shortly after administrated, and then the influence of 14T on the overall microbial community was mostly eliminated at 14 dpi.

**Fig. 3:**
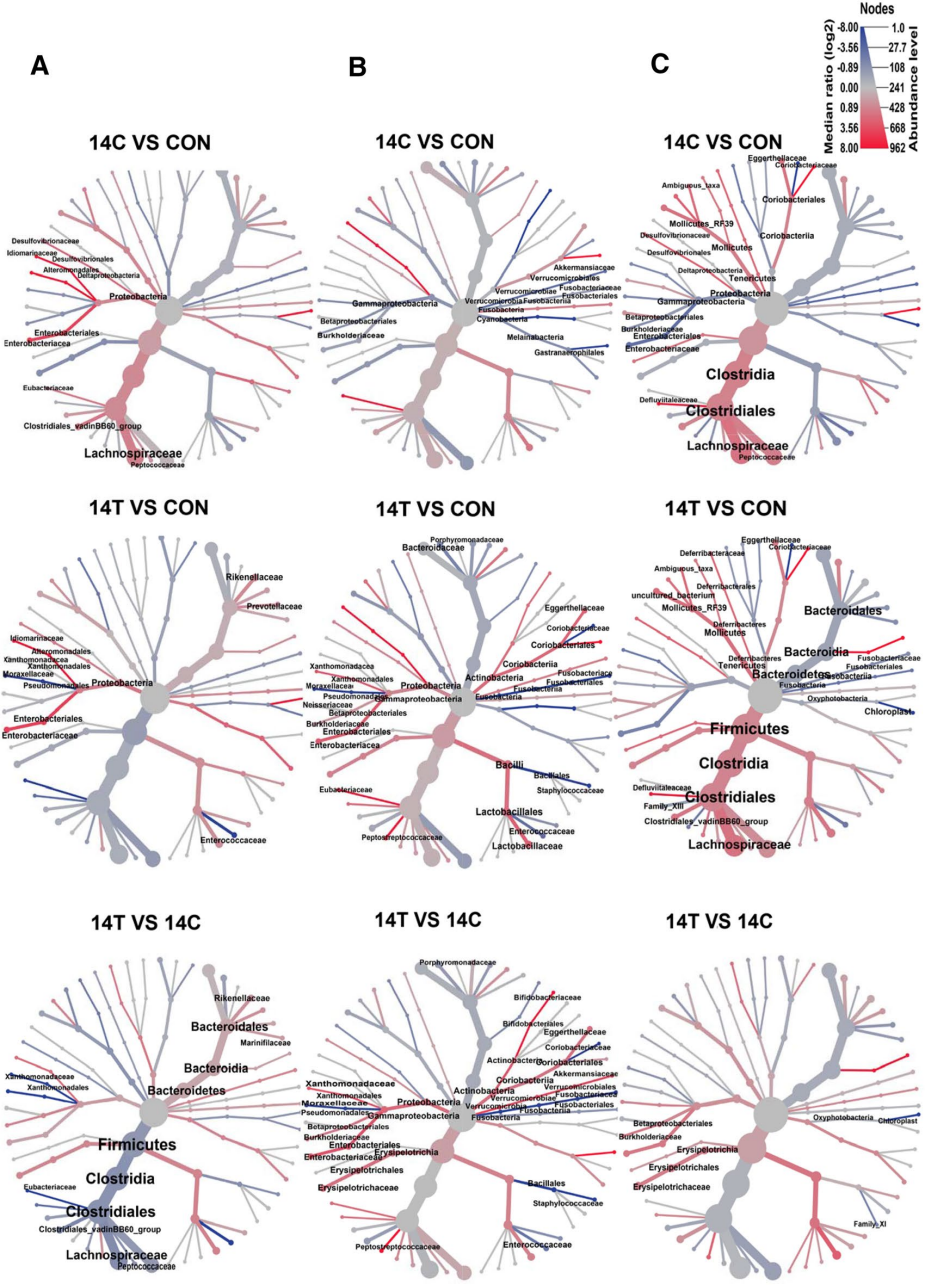
Heat tree of differentially abundant taxa in gut microbiota classified at family level (panel **A**, 0 dpi; panel **B**, 2 dpi; panel **C**, 14 dpi). The color of each taxon represents the log2 ratio of median proportion of reads observed in a given treatment. Only significantly differentially abundant taxa were labeled, which were determined by Heat_tree function in the Metacoder package implemented in MicrobiomeAnalyst server. Width of nodes and edges denotes the relative abundance of the given taxa. Ten samples from 14T and 14C treatments, and nine samples from the control group were included in this analysis

Through group-wise comparisons, we observed that several taxa, such as *Enterobacteriaceae*, exhibited consistent dynamic alteration in relative abundance in both 14T and 14C groups compared with controls (determined by the Heat_tree function in Metacoder package implemented in MicrobiomeAnalyst, adjusted *p* < 0.05, same herein) (Fig. 3 and Additional file 2: Table S2). The relative abundance increases of *Enterobacteriaceae* in 14T and 14C groups at 0 dpi were mainly contributed by the administrated *S*. Derby strains (accounting for 70.8% of the identified *Enterobacteriaceae*), while the contribution of the administrated strains to the *Enterobacteriaceae* composition was small (< 1%) at 2 dpi and 14 dpi. Interestingly, quite a few taxa were found to exhibit distinct alteration in relative abundance for 14T- and 14C-treated mice compared with control ones (Fig. 3 and Additional file 2: Table S2). At 0 dpi, several taxa affiliated with *Clostridiales* (an order affiliated with Firmicutes) exhibited significantly increased relative abundance in 14C-treated mice, but not in the 14T-treated mice compared with the control. At the same time, the relative abundance of *Rikenellaceae* and *Prevotellaceae*, both of which are affiliated with Bacteroidetes, was dramatically increased in 14T-treated mice, but not in 14C-treated mice, compared with the control. Significant differences in relative abundance of Firmicutes and Bacteroidetes between the 14T and 14C groups were also observed (Fig. 3A and Additional file 2: Table S2). Several taxa that are not affiliated with Firmicutes and Bacteroidetes, such as *Burkholderiaceae* (affiliated with Proteobacteria) and *Bifidobacteriaceae* (affiliated with Actinobacteria), were observed to exhibit differential relative abundance between 14C and control or 14T and control groups at 2 dpi, with little overlap observed between the majority of these differentially abundant taxa in the two group-wise comparisons (Fig. 3B and Additional file 2: Table S2). Of note, the relative abundance of *Lactobacillaceae*, *Bifidobacteriaceae*, *Enterobacteriaceae*, and *Akkermansiaceae*, were found to be higher in the 14T group compared with the 14C group (Fig. 3B and Additional file 2: Table S2). At 14 dpi, the bacterial community of the 14T- and 14C-treated mice trended to show similar composition with many fewer differentially abundant taxa observed compared with 2 dpi (Figs. 3B and C and Additional file 2: Table S2); however, the community in both groups diverged from that of the control group, suggesting that the infection of two strains caused dysbiosis of the gut microbiota, although the majority of the infected *S.* Derby strains were eliminated by 14 dpi.

### Strains 14T and 14C exhibited different response to mice-originated antagonistic *Ligilactobacillus* strains

**Fig. 4:**
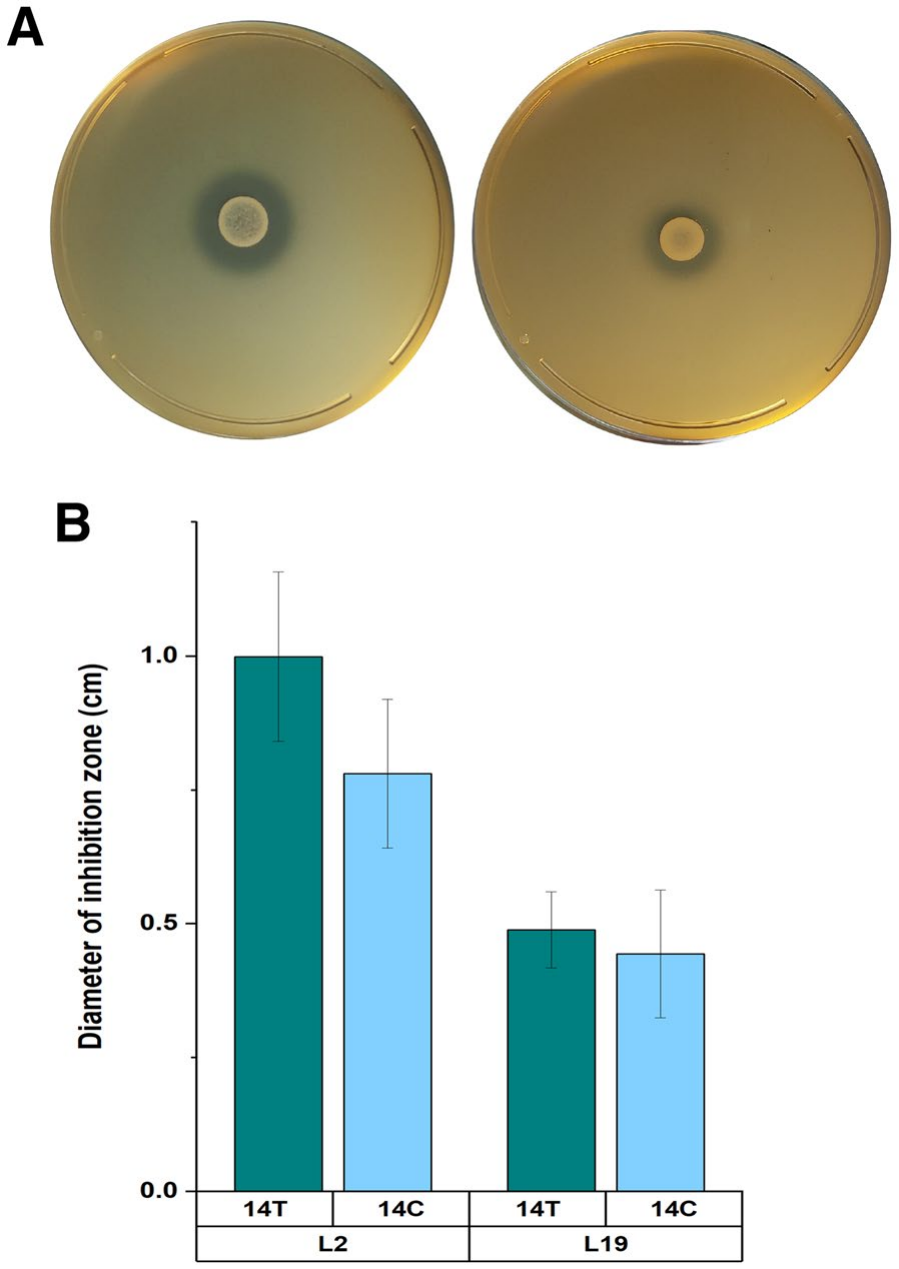
**A** Antagonistic activity of *Lactobacillus murine* isolates against *Salmonella* Derby 14T (left) and 14C (right). **B** Diameter of the inhibition zone of *Lactobacillus murine* isolates against 14T and 14C, shown as mean ± SD, n = 3

Through microbiota profiling analysis, we found that *Lactobacillaceae* exhibited increased relative abundance in both 14T- and 14C-treated mice compared with the control mice at 2 dpi; moreover, this taxon consistently exhibited higher, although not significantly, relative abundance in the 14T group than in the 14C group during the course of infection (Fig. 3 and Additional file 2: Table S2). *Lactobacillus* (a single genus based on Silva 132 database, has been reclassified to several genera since 2020) was the most predominated genus affiliated with *Lactobacillaceae*, and accounted for 99.8% of the accumulated relative abundance of *Lactobacillaceae*. Given that many members affiliated with *Lactobacillus* are commonly known as probiotic bacteria [23], the *Lactobacillus* bacteria were isolated from the fecal samples of treated mice using MRS medium. Two representative isolates, L2 and L19, both of which were affiliated with *Ligilactobacillus murinus*, were obtained. Interestingly, both L2 and L19 strains exhibited stronger, although not significantly, inhibitory effects on growth of *S.* Derby 14T compared with 14C (t-test, *p* > 0.05 for both strains). The zone of inhibition against 14T and 14C was 1.0 ± 0.16 cm (mean ± SD) and 0.78 ± 0.14 cm (28% higher) for L2, and the inhibition zone against 14T was 0.49 ± 0.07 cm and the value against 14C was 0.44 ± 0.12 cm (10% higher) for L19 strain, respectively (Fig. 4).

## Discussion

In this study, we investigated gut microbiota alteration patterns triggered by infection with two *S*. Derby strains from the same ST40 group but with distinct epidemic patterns (i.e., rarely-distributed vs. prevalent). Our results demonstrated that infection with *S.* Derby strains triggered distinct microbiota responses compared with that of well-studied *S.* Typhimurium. The diversity of gut microbiota was increased in *S.* Derby-treated mice, which was opposite compared with the *S.* Typhimurium-treated mice [24–26]. High diversity of gut microbiota can benefit the host by providing nutrients as well as resisting against pathogen colonization; for instance, higher complexity in gut microbiota usually results in increased protection against *Salmonella*-induced gut inflammation [27]. Certain consistent altered patterns were observed in the gut microbiota of mice infected with *S.* Derby and *S.* Typhimurium, such as reduced relative abundance of *Prevotella* and *Odoribacter* [10]; however, the taxonomic composition was strikingly different. For example, infection with *S.* Typhimurium significantly increased the relative abundance of *Citrobacter*, a potentially pathogenic bacteria [25, 28]*,* whereas infection with both 14T and 14C strains decreased the relative abundance of *Citrobacter* (Additional file 1: Figure S4). The microbiota composition is strongly associated with the susceptibility to enteric pathogen infection [27, 29]. The differences in microbiota alteration between the *S.* Typhimurium- and *S*. Derby-infected mice and its association with the consequences of the two serotypes on the hosts need to be further explored.

Interestingly, although strains *S*. Derby 14T and 14C used in this study shared highly conserved genomic contents [14, 15], the host symptoms and gut microbiota alteration patterns mediated by infection with the two strains were distinct (Figs. 1, 2, 3). Strain 14T treatment triggered a stronger inflammation response compared with 14C, but a smaller spleen-translocated 14T population was observed. This observation was in concert with the host cell adhesion results in that fewer 14T cells adhered to MC 38 cells compared with 14C (Fig. 1). Low-grade inflammation may benefit 14C, possibly by making a trade-off between inflammation and dissemination to and/or survival in the liver and spleen [29]. Of note, the host inflammation response was measured based on the Lipocalin-2 assay in this study, and the result might represent only a part of the immune response. Shortly after intragastric administration (4 h after treatment), the microbial diversity of 14C-treated mice was increased, whereas that of 14T-treated mice was decreased (Fig. 2). Higher complexity of gut microbiota is believed to be associated with increased protection against *Salmonella*-induced gut inflammation [27]. At 0 dpi, *Enterobacteriaceae* exhibited increased relative abundance in both 14T and 14C treated mice compared with the control mice at 0 dpi, which was mainly attributed to the administrated *S*. Derby strains; however, the high population of *S*. Derby in the gut microbiota was temporary and represented a very minute fraction of the gut microbiota at 2 dpi and 14 dpi. Increased relative abundance of *Clostridiales* in 14C-treated mice, and higher relative abundance of *Rikenellaceae* and *Prevotellaceae* in 14T-treated mice were also observed at 0 dpi. Members of *Clostridiales* are known as short chain fatty acids producers, which can down-regulate the expression of virulence genes of *Salmonella*, as well as limit O_2_ availability in the lumen of the gut, together restricting the expansion of *Salmonella* [4, 30, 31]. In contrast, *Rikenellaceae* and *Prevotellaceae* produce hydrogen, which may benefit *Salmonella* in terms of expansion and colonization at the initial infection stage [32, 33]. At 2 dpi, the relative abundance of *Lactobacillaceae*, *Bifidobacteriaceae*, *Enterobacteriaceae*, and *Akkermansiaceae*, were found to be dramatically higher in 14T-treated mice compared with 14C-treated mice (Fig. 3). *Lactobacillaceae* may reduce *Salmonella* shedding and translocation to liver and spleen [34–37]. *Enterobacteriaceae,* population of which was increased as driven by the elevated oxygen level in the gut due to enteric pathogens infection [38], were reported to restrict *Salmonella* expansion and infection by competing for iron, oxygen and other resources with this pathogen [24, 39, 40]. *Akkermansiaceae* are known to exacerbate inflammation in *S.* Typhimurium-infected gnotobiotic mice [41]. Furthermore, our results demonstrated that identical microbiota members can exhibit different direct-contact antagonistic activities against 14T and 14C (Fig. 4). Thus, we speculate that the more aggressive 14T strain triggered higher levels of host and microbiota immune responses, which hindered 14T from translocating to spleen and liver, resulting in lower 14T in vivo populations and restricted distribution compared with 14C.

## Conclusion

Overall, our study revealed distinct and conserved host gut microbiota response patterns triggered by the two *S.* Derby strains, 14T and 14C, which represented two distinct epidemic sub-populations with conserved genomic background. Colonization resistance conferred by the microbiota helps the host resist a variety of pathogens including *Salmonella*. Elucidating the differences and conservations of gut microbiota–*Salmonella* interactions at serotype- and strain-level will help us understand the epidemic differences in *Salmonella*, and benefit the development of microbiome engineering-based therapies.

## Methods

### *Salmonella* strains

Two representative *S.* Derby strains, 14T-T8N3 and 14C-D14P2 (*S.* Derby 14T and 14C herein), were used in this study. The two strains were isolated from pork samples collected in Yangzhou, Jiangsu, China, and affiliated with *Salmonella* sequence type (ST) 40 by multilocus sequence typing analysis, but represented two sub-populations with different population sizes (14T from the distribution-restricted CRISPR-type 39 and 14C from the prevalent CRISPR-type 38 sub-populations) (Additional file 1: Figure S1) [14, 15]. The strains were grown in Luria–Bertani (LB) broth with shaking at 37 ℃ overnight, and the bacterial population was adjusted to OD_600_ = 1.0 with PBS, then centrifuged and suspended with 2.5% NaHCO_3_ solution for intragastric administration.

### Cellular adhesion assays

The MC38 (Mouse colon cancer epithelial cell) cell line was purchased from Hunan Fenghui Biotechnology Co., Ltd. (Hunan, China) (catalog no. CL0203). *S.* Derby 14T and 14C strains were cultured in LB medium at 37 °C for 16 h, and then diluted into a new LB medium and incubated to OD_600_ = 1.0. The MC38 cells were seeded into 24-well plates with 4 × 10^5^ cells per well, and cultured overnight at 37 °C with 5% CO_2_. A total of 1 mL of bacterial culture was collected and washed twice with Dulbecco’s Modified Eagle Medium (DMEM) (Gibco, Grand Island, NY, USA), and then added to each well with a multiplicity of infection (MOI) of 20:1. The cells were incubated at 37 °C for 1 h. Then the cultured cells were washed twice with DPBS (Gibco, Grand Island, NY, USA), and lysed with 0.1% Triton X-100. The lysates were serially diluted and the appropriate dilutions were coated on the LB plates to calculate the number of bacteria. This experiment was performed four times independently, with three technical replicates used each time.

### Animals and experimental design

To select representative time points during the infection course for gut microbiota analysis, we firstly determined the in vivo* S*. Derby population dynamic in the infected mice. The more virulent strain 14T was used in this assay. Thirty-five 6-weeks old female C57BL/6J mice were purchased from Beijing Vital River Laboratory Animals Technology Co., Ltd. The mice were divided into 7 groups randomly, each group contains 5 mice. One group of mice were sacrificed and the spleen, liver, duodenum, ileum, colon and cecum tissues were collected one day before *S*. Derby oral administration. Then the remaining mice were orally administered 2 × 10^8^ CFU (200 μL) of *S.* Derby 14T strain. The treated mice were sacrificed at 0 dpi (4 h after administration), 2 dpi, 4 dpi, 7 dpi, 10 dpi and 12 dpi, one group of mice at each time point, and the organ tissues were collected accordingly. The *Salmonella* population in the collected organ tissues were determined as described by Zhou et al. [42].

To investigate the gut microbiota responses to *S*. Derby infection, thirty 6-weeks old female C57BL/6J mice were used. The mice were divided randomly into 3 groups with 10 mice in each group, with 5 mice housed in one cage. Mice were orally administered 2 × 10^8^ CFU (200 μL) of *S.* Derby strain 14T or 14C, or administered an equal volume of 2.5% NaHCO_3_ solution to serve as controls.

All mice were housed in isolators and kept in a room with controlled temperature, light, and ventilation. SPF-grade chows (Jiangsu xietong Bioengineering Co., Ltd.) and sterile water were provided to the mice. The body weight of each mouse was measured daily during the course of infection, with the baseline weight measured on the day prior to treatment (−1 dpi). The body weight dynamic of treated mice was calculated as: weight measured at day n post infection (Dn)/baseline. Fecal samples were collected from all mice on 0 dpi (4 h post infection), 2 dpi, and 14 dpi. Fecal pellets collected from individual mouse were measured and stored at −70 ℃ until further processing.

### Inflammation marker quantification

Lipocalin-2 is known as a good biomarker of inflammation [43]. Lipocalin-2 levels in the serum samples were measured using the Duoset murine Lcn-2 ELISA kit (R&D Systems, Minneapolis, MN, USA) according to the manufacturer’s manual. Briefly, blood samples collected by immediate postmortem cardiac puncture were placed at 4 ℃ overnight, then centrifuged at 4500 rpm for 5 min; the supernatant was transferred to a new 1.5 mL microtube, and centrifuged again for another 5 min at 4500 rpm. The supernatant serum was stored at −20 ℃ until use. For lipocalin-2 quantification, the serum samples were diluted properly.

### 16S rRNA gene sequencing and analysis

The microbiome DNA was extracted from fecal samples using the PureLink Microbiome DNA Purification kit (Invitrogen, Carlsbad, CA, USA) according to the manufacturer’s instructions. The quality of the extracted DNA was checked using a NanoDrop spectrophotometer (Thermo Scientific, Carlsbad, CA, USA). The v4 region of the 16S rRNA gene was amplified using the 515F-806R primer set and sequenced on an Ion S5™ XL platform (Thermo Scientific, Carlsbad, CA, USA) by Novogene, China. The raw reads generated by the Ion S5™ XL platform were quality checked and filtered using an in-house pipeline by Novogene. Clean data were further analyzed using the Amplicon-based Analysis workflow implemented in Microbial Genomics module, CLC Genomics Workbench (ver. 20) with default parameters, in which low-quality and chimeric reads were removed, and the remaining reads were clustered to operational taxonomic units (OTUs) at 97% similarity using a reference-based (Silva 132 [44]) approach. The generated OTU table was then uploaded to Microbiomeanalyst server for further analysis [45] with the feature filter step re-set as Minimum count 2, Prevalence in samples (%) 10, percentage in samples (%) 5, and other parameters set as default. The alpha diversity indices (Shannon and Chao1) and beta diversity metrics (Bray–Curtis) were calculated using Microbiomeanalyst server. Beta diversity metrics were visualized using principal coordinate analysis (PCoA) based on the Bray–Curtis indices, and treatment-dependent differences in beta diversity were tested using multivariate permutational ANOVA (PERMANOVA) based on the Bray–Curtis similarities. Pairwise comparisons of communities across the three treatments at 0 dpi, 2 dpi and 14 dpi were performed and visualized using the Heat_tree function in the Metacoder package implemented in MicrobiomeAnalyst server, in which the differentially abundant taxa are determined by a Wilcoxon rank-sum test followed by Benjamini–Hochberg (FDR) correction [46].

### Isolation of fecal lactic acid bacteria strains

The fecal samples were collected, and homogenized with 0.85% saline solution. Serial tenfold dilution was performed and 100 μL aliquots were plated on MRS agar. Plates were incubated at 37 ℃ for 48 h. Three to five colonies selected from each plate were streaked at least three times on MRS agar plates, and then colonies were cultured in 10 mL MRS broth. DNA was extracted from overnight cultures, and 16S rRNA gene was amplified using primer set 27F and 1492R. PCR products were submitted to Genscript (Nanjing, China) and Sanger sequenced for taxonomy identification. All isolates were sub-cultured in MRS broth and stored at −70 ℃ for future experiments.

### Antagonistic activity of isolated *Ligilactobacillus* strains against *S.* Derby 14T and 14C

Antagonistic activity against the two *S.* Derby strains of the isolated *Ligilactobacillus* strains was measured using the agar spot method as described by Toure and Koohestani [47, 48] with modifications. Briefly, 10 μL of 24 h culture of a *Ligilactobacillus* strain was spotted in the middle of the MRS agar plate, and incubated at 37 ℃ for 24 h. Then the incubated plates were overlaid with 10 mL of LB containing 0.75% agar at 45 ℃, seeded with 1% (v/v) of active overnight cultured of *S.* Derby strain 14T or 14C (final concentration 10^6^ CFU mL^−1^), and incubated aerobically at 37 ℃ for another 12–16 h. Diameter of the inhibition zone was measured and recorded. This assay was repeated 3 times, and three technical replicates were used each time.

## Supplementary Information

**Additional file 1: Figure S1.** Minimum spanning tree analysis of CRISPRS types for Salmonella Derby. The CRISPR type 38 and type 39 were yellow and blue colored, respectively. **Figure S2.** Rarefaction curve of all samples. The x axis represents the sequencing data and the y axis represents the OTU numbers in the order of the x axis. **Figure S3.** Principal coordinate analysis showing the beta diversity between treatments, as determined using Bray–Curtis similarities. Results of PERMANOVA are given on each plot; legend at right. **Figure S4.** The relative abundance comparison of Prevotella (A), Odoribacter (B), and Citrobacter (C) among the 14T, 14C and control mice. The relative abundance of the three genera was significantly higher in control mice compared with 14T- or 14C-treated mice as determined by Heat_tree function in the Metacoder package implemented in MicrobiomeAnalyst server. **Table S1.** The population dynamic of S. Derby in different organs of the orally administrated mice.

**Additional file 2: Table S2.** Relative abundance comparison across the three treatments. The differentially abundant taxa were determined at family level and labeled accrodingly.

## Data Availability

The 16S sequencing data have been deposited in China National GenBank database (CNGBdb) under project ID CNP0001062.
