# Supplementary material for: Closely related Salmonella Derby strains triggered distinct gut microbiota alteration
Source: Gut Pathog. 2022 Jan 25;14:6. doi: 10.1186/s13099-022-00480-6 (PMC8787955; doi:10.1186/s13099-022-00480-6)
Supplement: Supplementary file 1 — Additional file 1: Figure S1. Minimum spanning tree analysis of CRISPRS types for Salmonella Derby. The CRISPR type 38 and type 39 were yellow and blue colored, respectively. Figure S2. Rarefaction curve of all samples. The x axis represents the sequencing data and the y axis represents the OTU numbers in the order of the x axis. Figure S3. Principal coordinate analysis showing the beta diversity between treatments, as determined using Bray–Curtis similarities. Results of PERMANOVA are given on each plot; legend at right. Figure S4. The relative abundance comparison of Prevotella (A), Odoribacter (B), and Citrobacter (C) among the 14T, 14C and control mice. The relative abundance of the three genera was significantly higher in control mice compared with 14T- or 14C-treated mice as determined by Heat_tree function in the Metacoder package implemented in MicrobiomeAnalyst server. Table S1. The population dynamic of S. Derby in different organs of the orally administrated mice. [file 13099_2022_480_MOESM1_ESM.docx]

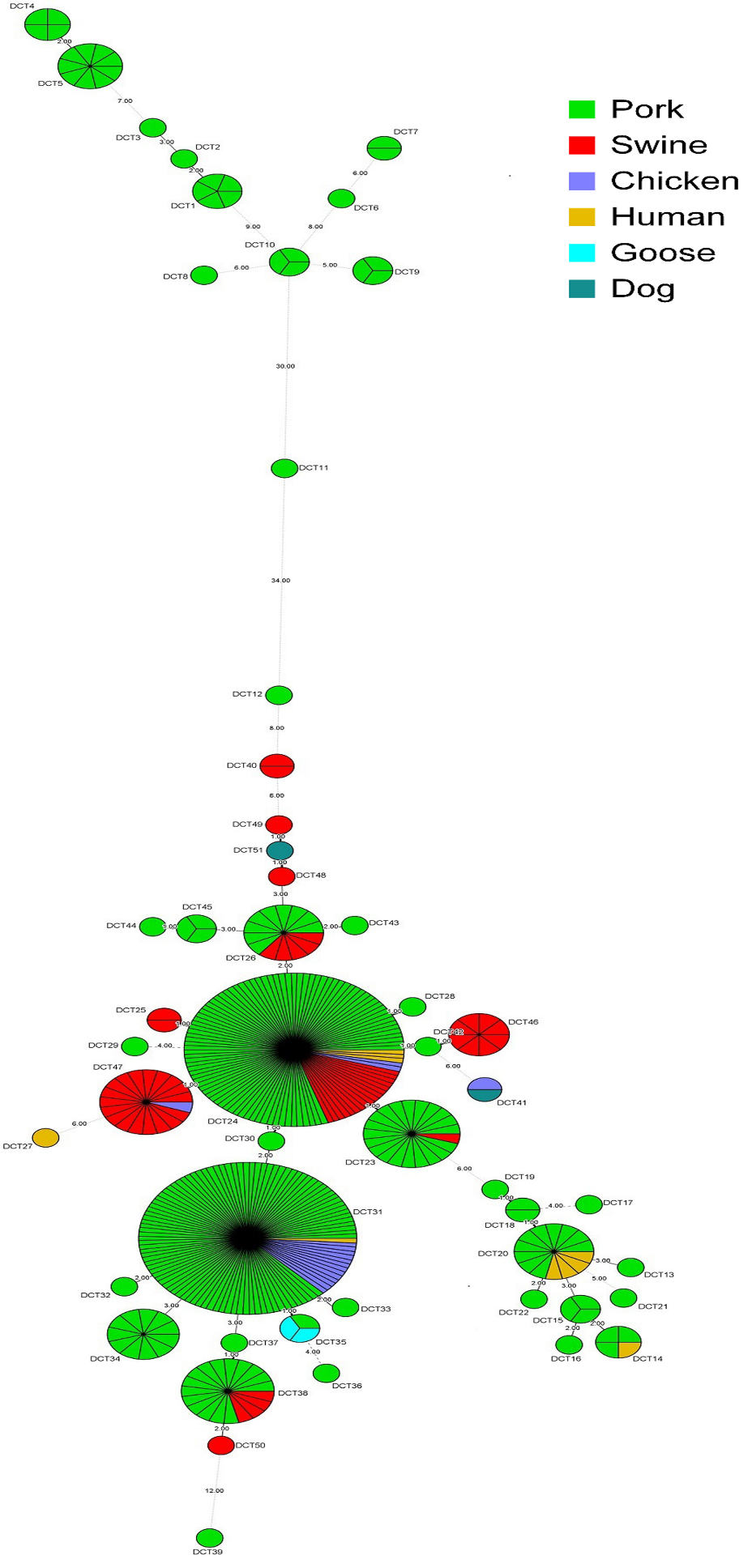


Figure S1. Minimum spanning tree analysis of CRISPRS types for *Salmonella* Derby. The CRISPR type 38 and type 39 were yellow and blue colored, respectively.


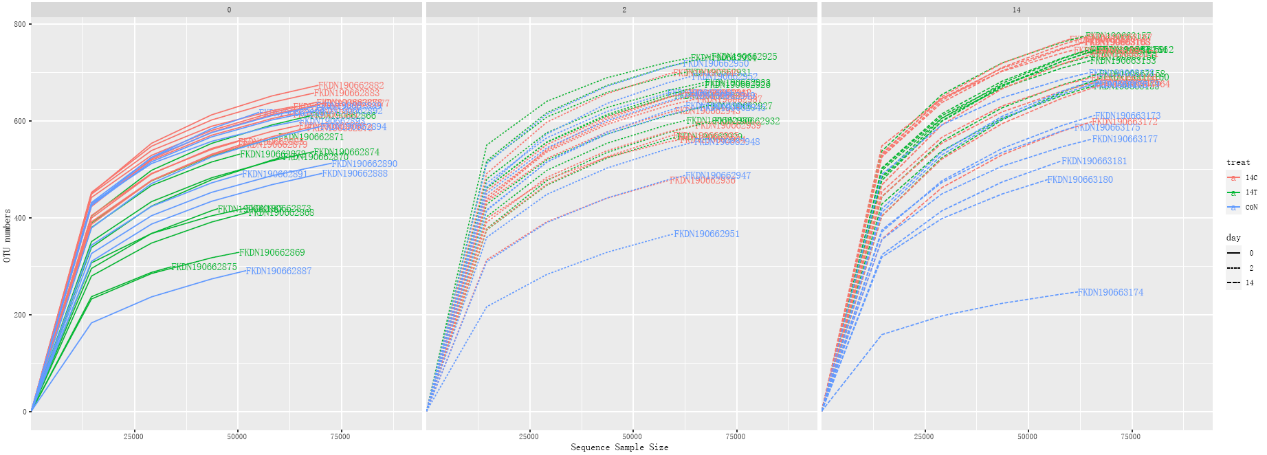


Figure S2. Rarefaction curve of all samples. The x axis represents the sequencing data and the y axis represents the OTU numbers in the order of the x axis.


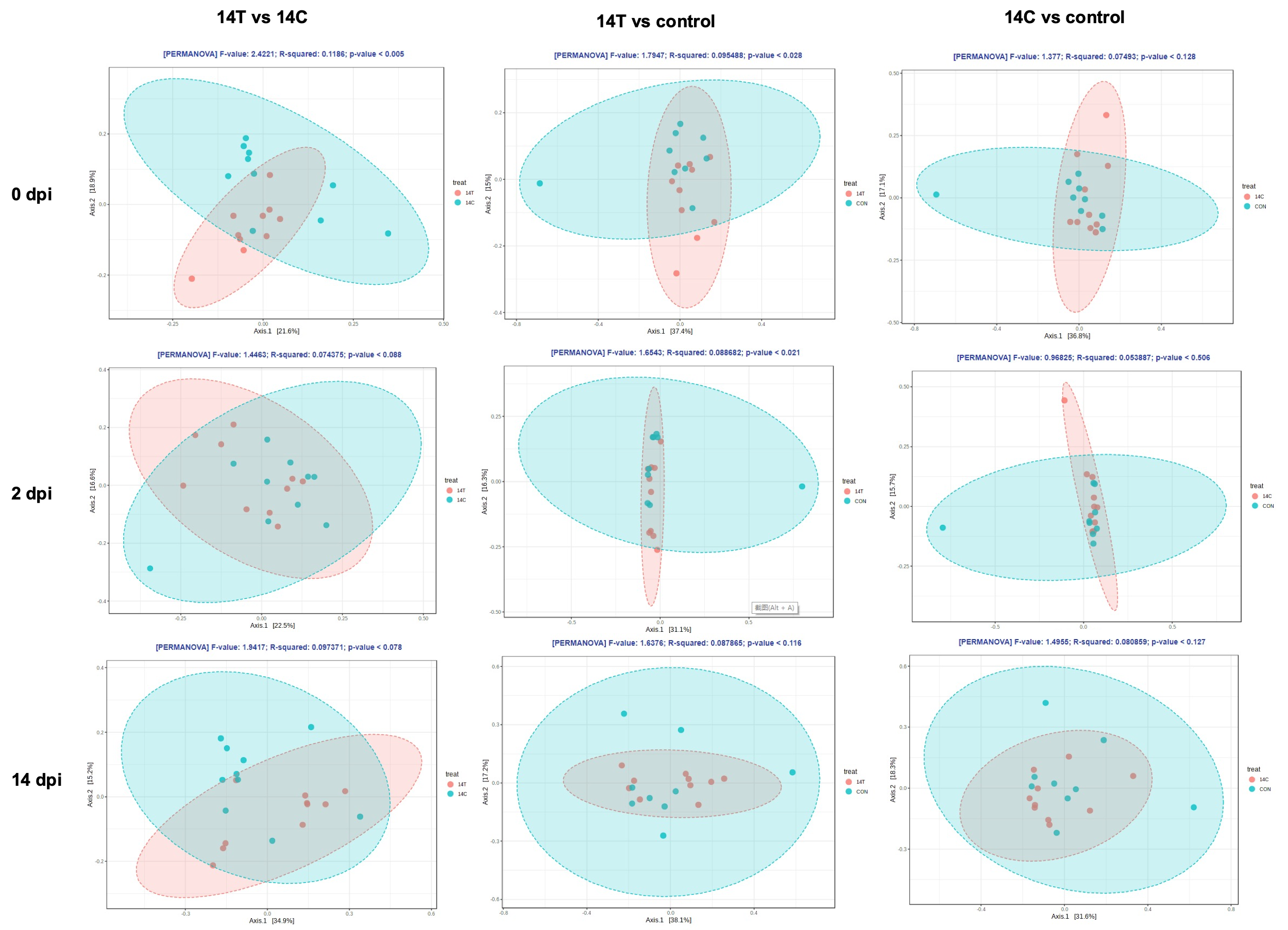


Figure S3. Principal coordinate analysis showing the beta diversity between treatments, as determined using Bray–Curtis similarities. Results of PERMANOVA are given on each plot; legend at right.


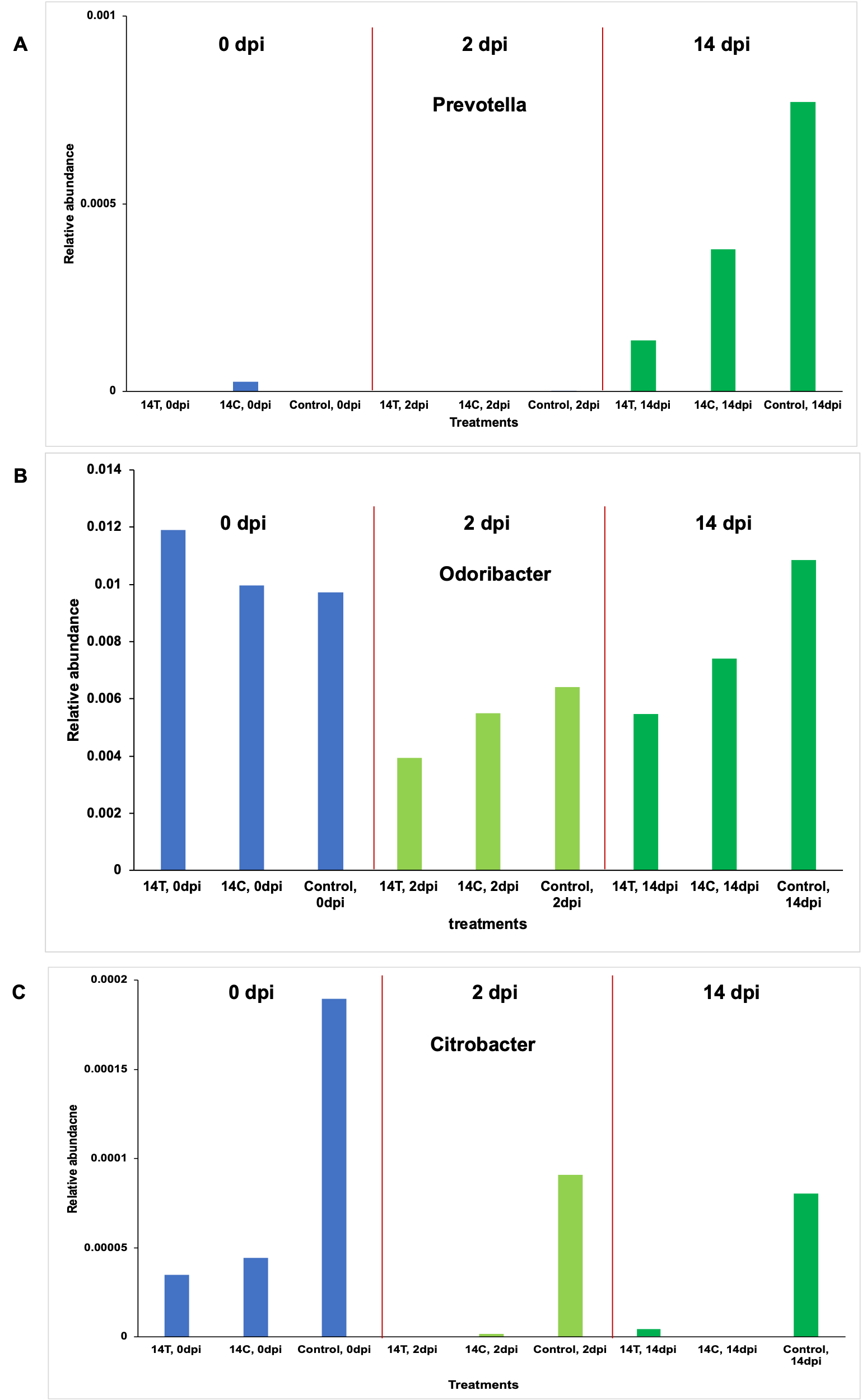


Figure S4. The relative abundance comparison of *Prevotella* (A), *Odoribacter* (B), and *Citrobacter* (C) among the 14T, 14C and control mice. The relative abundance of the three genera was significantly higher in control mice compared with 14T- or 14C-treated mice as determined by Heat_tree function in the Metacoder package implemented in MicrobiomeAnalyst server.

Table S1. The population dynamic of *S*. Derby in different organs of the orally administrated mice.

| Sample | Organ | Infection time | | | | | | |
| --- | --- | --- | --- | --- | --- | --- | --- | --- |
|  |  | -1 dpi | 0 dpi | 2 dpi | 4 dpi | 7 dpi | 10 dpi | 12 dpi |
| A1 | Spleen | BD | BD | 2.9 | BD | 3.6 | BD | 3.6 |
| A2 |  | BD | BD | 4.0 | BD | BD | 3.4 | BD |
| A3 |  | BD | BD | 3.2 | 4.0 | BD | 3.1 | BD |
| A4 |  | BD | BD | BD | 3.3 | 2.9 | BD | 3.1 |
| A5 |  | BD | BD | 5.7 | BD | BD | BD | 3.8 |
| A1 | Liver | BD | BD | BD | BD | 2.6 | BD | BD |
| A2 |  | BD | BD | 2.1 | BD | BD | BD | BD |
| A3 |  | BD | BD | 2.8 | 2.6 | BD | BD | BD |
| A4 |  | BD | BD | BD | BD | BD | BD | BD |
| A5 |  | BD | BD | 5.9 | BD | BD | BD | 2.5 |
| A1 | Duodenum | BD | BD | 3.9 | BD | 4.7 | BD | 3.6 |
| A2 |  | BD | BD | 4.2 | BD | BD | BD | BD |
| A3 |  | BD | BD | 3.1 | 3.3 | 2.5 | BD | BD |
| A4 |  | BD | BD | BD | 3.2 | 3.3 | BD | BD |
| A5 |  | BD | BD | 5.8 | BD | BD | BD | BD |
| A1 | Ileum | BD | 4.9 | 5.1 | BD | 3.6 | BD | 4.9 |
| A2 |  | BD | 4.9 | 7.2 | 5.6 | BD | BD | BD |
| A3 |  | BD | 3.1 | 5.2 | 4.4 | 3.6 | 4.5 | BD |
| A4 |  | BD | 4.6 | BD | 4.6 | 3.3 | BD | BD |
| A5 |  | BD | 3.6 | 8.0 | BD | BD | BD | BD |
| A1 | Colon | BD | 3.4 | BD | BD | BD | BD | BD |
| A2 |  | BD | 3.4 | 3.9 | BD | BD | BD | BD |
| A3 |  | BD | 5.0 | BD | BD | BD | BD | BD |
| A4 |  | BD | 4.1 | BD | BD | BD | BD | 2.9 |
| A5 |  | BD | BD | 7.6 | BD | BD | BD | BD |
| A1 | Cecum | BD | 4.8 | 0.7 | BD | 4.2 | BD | BD |
| A2 |  | BD | 5.2 | 0.7 | 3.9 | BD | 2.9 | BD |
| A3 |  | BD | 4.4 | BD | BD | 2.7 | 3.5 | BD |
| A4 |  | BD | 4.7 | 0.7 | 5.0 | BD | BD | 2.8 |
| A5 |  | BD | 3.6 | 0.6 | BD | 2.4 | BD | 2.3 |

The average population was shown as log_10_ CFU/gram organ. BD, below the detection limit.
